# Surveillance Strategy for Barcelona Clinic Liver Cancer B Hepatocellular Carcinoma Achieving Complete Response: An Individualized Risk-Based Machine Learning Study

**DOI:** 10.3389/fbioe.2021.667641

**Published:** 2021-08-31

**Authors:** Qi-Feng Chen, Lin Dai, Ying Wu, Zilin Huang, Minshan Chen, Ming Zhao

**Affiliations:** ^1^ Department of Medical Imaging and Interventional Radiology, Sun Yat-sen University Cancer Center, Guangzhou, China; ^2^ State Key Laboratory of Oncology in South China, Guangzhou, China; ^3^ Collaborative Innovation Center for Cancer Medicine, Guangzhou, China; ^4^ Cancer Prevention Center, Sun Yat-sen University Cancer Center, Guangzhou, China; ^5^ Department of Liver Surgery, Sun Yat-sen University Cancer Center, Guangzhou, China

**Keywords:** Barcelona clinic liver cancer B, hepatocellular carcinoma, complete response, surveillance strategy, machine learning

## Abstract

**Background:** For patients with complete response (CR) of Barcelona Clinical Liver Cancer (BCLC) stage B hepatocellular carcinoma (HCC), there is no consensus regarding the monitoring strategy. Optimal surveillance strategies that can detect early progression of HCC within a limited visit after treatment have not yet been investigated. A retrospective, real-world study was conducted to investigate surveillance strategies for BCLC stage B HCC (BBHCC) patients with CR after curative treatment to support clinical decision making.

**Methods:** From January 2007 to December 2019, 546 BBHCC patients with CR after radical treatment were collected at Sun Yat-sen University Cancer Center. Seventy percent of patients were subjected to the train cohort randomly; the remaining patients comprised the validation cohort to verify the proposed arrangements. The random survival forest method was applied to calculate the disease progression hazard per month, and follow-up schedules were arranged to maximize the capability of progression detection at each visit. The primary endpoint of the study was the delayed-detection months for disease progression.

**Results:** The cumulative 1, 2, and 3-years risk-adjusted probabilities for the train/validation cohorts were 32.8%/33.7%, 54.0%/56.3%, and 64.0%/67.4%, respectively, with peaks around approximately the 9th month. The surveillance regime was primarily concentrated in the first year posttreatment. The delayed-detection months gradually decreased when the total follow-up times increased from 6 to 11. Compared with controls, our schedule reduced delayed detection. Typically, the benefits of our surveillance regimes were obvious when the patients were followed seven times according to our schedule. The optional schedules were 5, 7, 9, 11, 17, 23, and 30 months.

**Conclusion:** The proposed new surveillance schedule may provide a new perspective concerning follow-up for BBHCC patients with CR.

## Introduction

Hepatocellular carcinoma (HCC) was the 6th most diagnosed cancer type and the 4th leading cause of cancer death worldwide in 2018 (Bray et al., 2018). The Barcelona Clinical Liver Cancer (BCLC) algorithm is a useful HCC staging classifier that is utilized worldwide (Forner et al., 2018). The BCLC staging system has been extensively validated clinically, and it is the most commonly used system for HCC. Following BCLC guidelines, only early-stage patients (BCLC 0/A) should be treated with radical therapies (surgery/ablation). For BCLC B stage HCC (BBHCC) cases with large multifocal tumors and without vascular invasion or spread outside of the liver, transarterial chemoembolization (TACE) is recommended if liver function is maintained. Nevertheless, recent advances in technology and appropriate patient selection have gradually reduced the morbidity and mortality of radical treatments, which have been considered for BBHCC patients with promising results in terms of postoperative outcomes (Day et al., 2016; Chen et al., 2017). For example, Labgaa I et al. systematically analyzed 1,730 BBHCC patients and found that compared with TACE, surgery improved long-term survival; postoperative mortality was equivalent (Labgaa et al., 2020). In our previous study, ablation-TACE combination therapy had a better clinical efficacy than TACE monotherapy for BBHCC (Chen et al., 2017; Zhang et al., 2018). Therefore, selected BBHCC patients might benefit from radical therapies.

Regardless, follow-up is a confusing issue in the BCLC staging system, which is important for assessing treatment success and detecting disease progression. The practical monitoring strategies in guidelines are mainly based on expert opinions. It is recommended that cancer survivors should be monitored regularly after treatment (radiological examination three to 6 months on average) to expedite detection of disease progression (Kanwal and Singal, 2019; Chen et al., 2020a). Tumors may relapse after radical therapies, leading to an early diagnosis of tumor relapse being more likely to be treated curatively, which can better manage the disease and prolong survival (Trinchet et al., 2011; Vogel et al., 2018). At present, the question of what is the best monitoring strategy that can detect tumor progression in a timely manner after treatment remains. Although recent guidelines suggest follow-up strategies for monitoring after curative treatment (Chen et al., 2020a), there is a lack of a specific surveillance algorithm for curatively treated HCCs, especially for BBHCC patients who show a complete response (CR) after radical treatment. The guidelines do not recommend specific monitoring intervals for BBHCC patients with CR, cases that are more complicated and likely to relapse earlier than BCLC stage 0/A cases. In addition, it remains unclear whether the current surveillance strategies are adequate.

In this study, we applied a random survival forest (RSF) analysis, a machine learning method, to calculate the probability of disease progression for each month. Thereafter, a risk-associated surveillance program was established on the basis of the abovementioned disease progression probabilities. The surveillance regime was evaluated by calculating the total number of delayed-detection days, followed by comparison to other surveillance proposals. Our surveillance strategy for BBHCC patients with CR after radical therapy will support clinical follow-up decision making.

## Materials and Methods

### Patient Datasets and Processing

We retrospectively collected BBHCC patients who underwent radical treatment (surgery/ablation) from an institutional database at Sun Yat-sen University Cancer Center from January 2007 to December 2019. All cases were diagnosed with HCC according to pathology or clinical criteria (Xie et al., 2020). A total of 2,193 consecutive BBHCC patients were initially eligible. This study included BBHCC patients who received radical treatment and achieved CR. Patients underwent multidetector computed tomography (CT) and/or magnetic resonance imaging (MRI) routinely to evaluate the local or distant extension of the primary tumors. After radical treatment, the patients were instructed to undergo multiphasic cross-sectional chest, abdomen, and pelvis high-quality imaging checks within first month, and every 2–6 months thereafter. CR is defined as no disease progression (death or local/distant tumor progression) during first follow-up after radical treatment. We excluded patients who had any of the following criteria: <18 or >80 years, mixed liver cancer, or death due to postoperative complications. Clinical and blood tests were performed at diagnosis and surveillance. After excluding 1,639 patients according to the exclusion criteria, 546 patients were included in the study. All patients received radical treatment, with some being treated with TACE (considered noncurative treatment) before radical treatment. The train cohort consisted of 382 patients (70%); 164 patients (30%) were used as the validation cohort. Considering the retrospective nature of the study, our cancer center institutional ethics committee approved the study protocol and waived the requirement for informed consent.

### Disease Progression Probability Calculation

To determine the optimal surveillance strategy, we first assessed the cumulative disease progression probabilities over 3 years in the two cohorts (train/validation cohorts) through the RSF method and calculated the probability of disease progression every month. As a machine learning tool, RSF can conduct right-censored survival statistical analysis (Taylor, 2011). The RSF method has a number of appealing features, with a major feature concerning our study being that none of the variables is deleted or selected, such that all of the variables influence the predicted result. In addition, RSF can incorporate situations in which the complex relationship between predictor and response variables occurs and predictors have nonlinear patterns and interactions. The RSF method plotted survival curves for two cohorts, involving all hazard-modified variables. Processing of the RSF method was conducted using the R package of random forest SRC.

### Development of a Risk-Based Follow-Up Schedule

After calculating the probability of disease progression for each month, a total number of follow-up times from the minimum of 6 (follow-up every 6 months) to the maximum of 11 (follow-up every 3 months from 4th month) was set. The follow-up times were assigned based on the progression probability of each month; the best strategy is to strike a balance between timely progression detection and minimal follow-up times.

We assessed the surveillance strategy based on the total delayed-detection days and compared it to a typical surveillance strategy (set as control), as follows: 7 times (Maas et al., 2020) (1, 3, 6, 9, 12, 18, 24, 30, and 36 months, which was put forward in a cooperative meeting of ECIO (European Conference on Interventional Oncology) and ESOI (European Society of Oncologic Imaging).

Therefore, within a 2-year period, the full supervision times for the hazard-based surveillance regime should be adjusted from a maximum of 11 to a minimum of 6. To explore an ideal follow-up schedule for rapidly revealing disease progression under minimal follow-up, we subsequently created a surveillance program covering a 3-years supervision ranging from 6 to 11 times. We assigned follow-ups to those months in which disease would more likely progress when one supervision was arranged for each month at most (Zhou et al., 2020).

### Delayed-Detection Calculation

Then, we compared our surveillance schedule with the control strategies. The capability of the supervision strategy was quantified by counting the total delayed-detection months in the train cohort. Delayed-detection months were defined as the time from disease progression to the next closest follow-up. As an example, if a patient progressed on the 200th day and the following most recent scheduled day was 240, then the delayed-detection days for that patient was 40. We calculated the total number of delayed-detection months for our plans and compared it with the control strategy. Strategy that reduced the sum of delayed-detection months with less follow-up time were considered preferable. The arrangements of the proposed schedule were also applied to patients in the validation cohort.

### Statistical Analysis

Disease progression was deemed death or local/distant tumor progression. Progression-free survival (PFS) was measured from the date of CR to disease progression or the last follow-up evaluation (August 2020). The χ2 or Fisher’s exact probability test was used for categorical variables. In the train group, the risk-based surveillance schedule was conducted by RSF. The time differences in delayed detection between our model and the recommended model were compared using the paired *t*-test or Kruskal-Wallis test. R language (version 3.6.0; R Foundation) was utilized for all analyses. A two-sided *p* < 0.05 indicated that the difference was of statistical significance.

## Results

### Patient Characteristics

**FIGURE 1:**
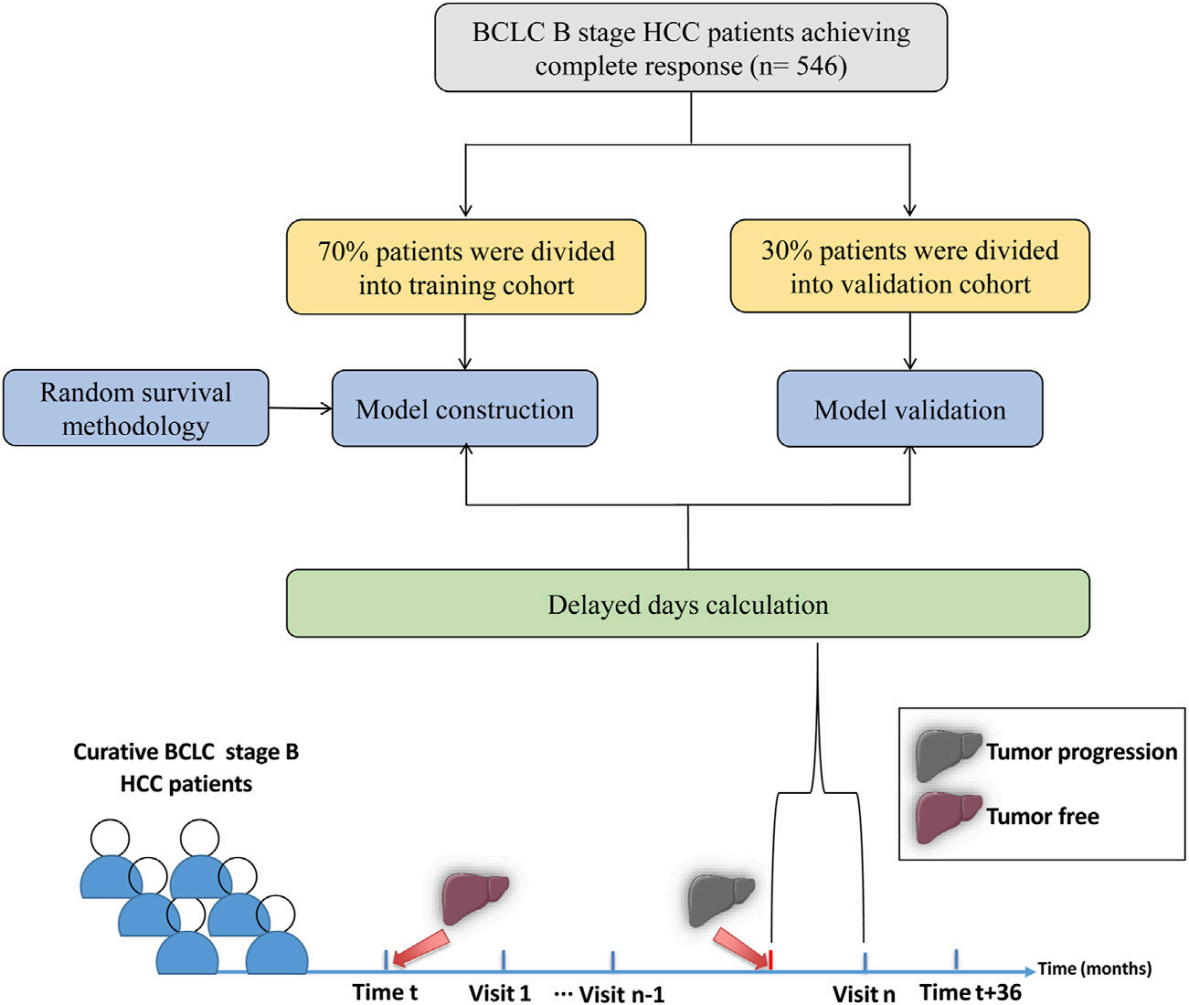
General design of the present study.

A total of 382 patients in the train cohort and 164 in the validation cohort were enrolled. The flowchart is illustrated in Figure 1. Table 1 shows the basic patient characteristics. Demographics were similar with regard to sex, age, hepatitis virus infection, α-fetoprotein (AFP) level, tumor size, tumors numbers, cirrhosis, combined TACE therapy, surgery or ablation treatment, tumor differentiation, satellite nodules, venous invasion, perineural invasion, capsule invasion, disease progression status, and PFS time between the train and validation cohorts (*p* > 0.05). In the whole cohort, progression occurred in 309 patients, with a median PFS of 13.7 months (interquartile range (IQR], 7.80–25.70 months).

**TABLE 1:** Patient characteristics in the train and validation cohorts.

	Overall (546)	Train (382)	Validation (164)	*p*-value
Gender = Male/Female (%)	480/66 (87.9/12.1)	338/44 (88.5/11.5)	142/22 (86.6/13.4)	0.631
Age (year) = <45/≥45 (%)	137/409 (25.1/74.9)	99/283 (25.9/74.1)	38/126 (23.2/76.8)	0.568
Hepatatis virus = No/Yes (%)	74/472 (13.6/86.4)	51/331 (13.4/86.6)	23/141 (14.0/86.0)	0.941
AFP(ng/ml) = <400/≥400 (%)	296/250 (54.2/45.8)	206/176 (53.9/46.1)	90/74 (54.9/45.1)	0.912
Tumor size = <50 mm/≥50 mm (%)	256/290 (46.9/53.1)	182/200 (47.6/52.4)	74/90 (45.1/54.9)	0.654
Tumor number = <4/≥4 (%)	458/88 (83.9/16.1)	318/64 (83.2/16.8)	140/24 (85.4/14.6)	0.624
Cirrhosis = No/Yes (%)	189/357 (34.6/65.4)	129/253 (33.8/66.2)	60/104 (36.6/63.4)	0.592
Combined TACE = No/Yes (%)	312/234 (57.1/42.9)	218/164 (57.1/42.9)	94/70 (57.3/42.7)	0.999
Surgery/Ablation (%)	436/110 (79.9/20.1)	307/75 (80.4/19.6)	129/35 (78.7/21.3)	0.734
Differentiation (%)	—	—	—	0.713
Well	175 (32.1)	118 (30.9)	57 (34.8)	—
Moderated	243 (44.5)	175 (45.8)	68 (41.5)	—
Poor	27 (4.9)	20 (5.2)	7 (4.3)	—
Unknown	101 (18.5)	69 (18.1)	32 (19.5)	—
Satellite nodules (%)	—	—	—	0.736
No	401 (73.4)	284 (74.3)	117 (71.3)	—
Yes	41 (7.5)	27 (7.1)	14 (8.5)	—
Unknown	104 (19.0)	71 (18.6)	33 (20.1)	—
Venous invasion (%)	—	—	—	0.753
No	303 (55.5)	216 (56.5)	87 (53.0)	—
Yes	139 (25.5)	95 (24.9)	44 (26.8)	—
Unknown	104 (19.0)	71 (18.6)	33 (20.1)	—
Perineural invasion (%)	—	—		0.487
No	439 (80.4)	308 (80.6)	131 (79.9)	—
Yes	3 (0.5)	3 (0.8)	0 (0.0)	—
Unknown	104 (19.0)	71 (18.6)	33 (20.1)	—
Capsule invasion (%)	—	—	—	0.879
No	188 (34.4)	133 (34.8)	55 (33.5)	—
Yes	255 (46.7)	179 (46.9)	76 (46.3)	—
Unknown	103 (18.9)	70 (18.3)	33 (20.1)	—
PFS = No/Yes (%)	237/309 (43.4/56.6)	167/215 (43.7/56.3)	70/94 (42.7/57.3)	0.897
PFS (median (IQR))	13.70 (7.80, 25.70)	14.15 (8.03, 25.35)	13.00 (7.47, 27.65)	0.572

TACE, transcatheter arterial chemoembolization; AFP, α-fetoprotein; PFS, progression-free survival; IQR, interquartile range.

### Calculation of Disease Progression Probability

**FIGURE 2:**
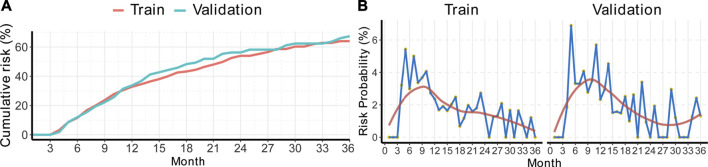
Cumulative risk curves and time-specific progression probabilities of BBHCC CR patients. **(A)** Cumulative risk curves. **(B)** Each month’s progression probability. BBHCC: Barcelona clinical liver cancer stage B hepatocellular carcinoma; CR: complete response.

To measure the monthly disease progression hazard of the train/validation cohorts, the monthly probability of disease progression was calculated by the RSF method, which was adjusted with clinical factors. Figure 2A shows the progression probabilities of patients in the train/validation cohorts. The cumulative 1, 2, and 3-years adjusted risk probabilities for the train cohort were 32.8, 54.0, and 64.0%, respectively, and those for the validation cohort were 33.7, 56.3, and 67.4%, respectively (Supplementary Table S1).

Then, the calculation of progression probability at a specific time was performed. The probability patterns in both the train/validation cohorts were quite similar. According to the data shown in Figure 2B, the disease progression incidence rose rapidly, reached a peak around approximately the 9th month, and decreased smoothly to a plateau less than 2% (Supplementary Table S2).

### Development of a Risk-Based Follow-Up Schedule

**FIGURE 3:**
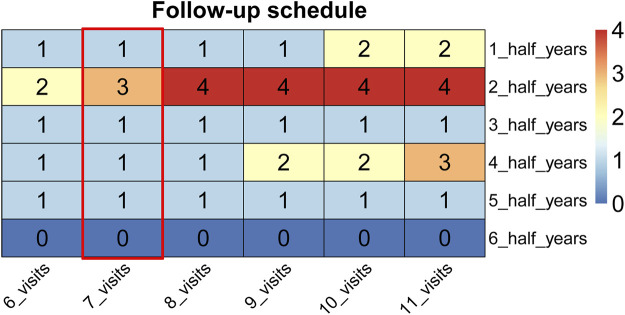
The supervision arrangements ranging from 6 to 11 follow-up times. The follow-up schedules of seven times are highlighted in the red box.

Next, a risk-based surveillance regime was established depending on the disease progression probability of each month by the prescribed method. The follow-up schedule with total follow-up times ranging from 6 to 11 for the first 3 years is depicted in Figure 3. The surveillance regime was concentrated primarily in the first year posttreatment with rather less supervision during the following years. The third year had relatively fewer follow-up times and more follow-up times were allocated in the second half year.

### Delayed Month Comparison

**FIGURE 4:**
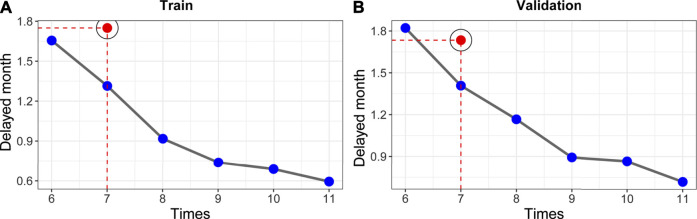
Establishment of risk-based surveillance arrangements. **(A,B)** In contrast with the control strategy (7 times, which was put forward in a cooperative meeting of ECIO and ESOI), our surveillance arrangements (blue points with gray curve connected) had fewer delayed-detection days.

We compared our model performance (the ability to detect disease progression in a timely manner) with that of controls (Figure 4). As shown in Figures 4A,B, the delayed-detection time gradually decreased when the total follow-up time increased from 6 to 11. The delayed-detection months of our surveillance regime (blue dots with gray curves connected) with that of the control (the red points indicated a 7 times follow-up strategy) were also compared. As presented in Figures 4A,B, under the same number of follow-up times, our monitoring arrangement significantly reduced the delayed-detection months, which was more efficient than in the controls. Typically, when patients were followed seven times according to our schedule, the advantage of our surveillance schedule was of significance.

**FIGURE 5:**
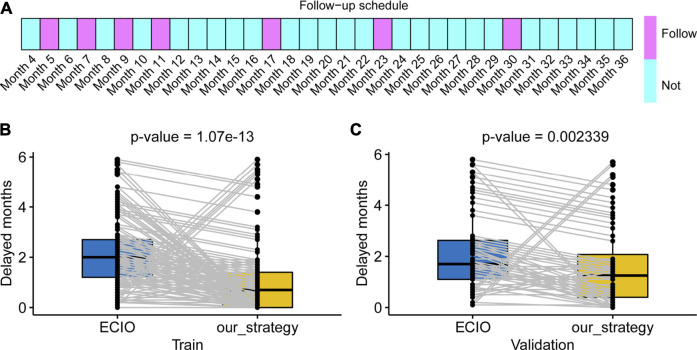
Our schedule and the clinical application. **(A)** The boxes panel show the months that should be followed. **(B,C)** Our schedule produces less delayed detection in both the train and validation cohorts. ECIO: European Conference on Interventional Oncology.

Our recommended supervision schedules are as follows. Our surveillance schedule involves seven times within 3 years (5, 7, 9, 11, 17, 23, and 30 months, respectively). The detailed schedule for each follow-up is shown in Figure 5A. In general, monitoring should be concentrated in the first year posttreatment. The proposed supervision schedules were further verified in individual disease progressed cases of train cohort and the validation cohort that had clinical characteristics that were almost consistent with those of the train cohort. For disease progressed patients, the surveillance strategy recommended by us significantly decreased the delayed-detection time compared with the control (Figures 5B,C, both *p*-values< 0.01).

## Discussion

Currently, there are limited validated data showing an optional surveillance schedule for BBHCC patients with CR. Given the potential for disease progression posttreatment, continued monitoring is necessary for these patients. In this population-based real-world study, we applied an RSF method to determine the risk of disease progression for each month. Thereafter, we propose a surveillance plan that is able to detect disease progression effectively at each follow-up. Typically, our model was more efficient than control schedules. Despite the fact that our project was generated using the BBHCC population, our risk-related monitoring method can be applied to develop monitoring strategies for other BCLC-stage HCC patients and can generally help develop personalized surveillance schedules after treatment.

Many professional associations have put forward guidelines for posttreatment management and have provided universal surveillance recommendations for HCC patients. However, most of these recommendations were derived from early HCC cases. Thus, due to the substantial differences in biology, therapy, and the way disease progresses, early HCC monitoring experience may not be applicable to BBHCC (Chen et al., 2019; Liu et al., 2020). Boas FE et al. enrolled 910 patients receiving 1,766 successive operations, including TACE, radioembolization, and ablation, at a single institution regardless of patient stage between 2006 and 2011 (Boas et al., 2015). Consistent with our results, they demonstrated that more recurrence occurred in the first year after treatment, leading to much more frequent screening in the first year. In april 2018, a joint session from ECIO and ESOI produced a recommendation based on the literature and expert opinion that the total number of follow-up times is 7 for liver-directed cases (first year: 1, 3, 6, 9, and 12 months; every 6 months thereafter) (Maas et al., 2020). However, the quality and quantity of evidence were limited. The recommendations conferred were based in part on expert opinion and consensus that applied to all liver cancer patients; moreover, it remains unknown whether the follow-up guidelines are most effective. As a result, we focused on BBHCC patients with CR and developed the present new superior surveillance strategy.

The purpose of tumor monitoring is to detect disease progression as early as possible (Wu et al., 2020a; Chen et al., 2020b). Thus, patients would ideally be checked every day, which is not practical in clinical practice. Regardless, the surveillance strategy can be improved based on disease progression probabilities per month by scheduling as close as possible surveillance time to the expected time. We assumed that the damage caused by delayed detection was proportionally associated with delayed detection. In fact, there might be a threshold value that could be useful for clinical decision making. When patients were followed within the threshold value, delayed-detection days could be ignored clinically. However, no published research solves this problem in a quantitative manner.

Currently, it is generally believed that the detection of disease progression as soon as possible is of utmost importance, as a number of cases of early progression can be treated effectively (Wu et al., 2020b; Li et al., 2020). Tsilimigras DI reported that 154 BCLC B/C patients underwent resection with an annual recurrence rate of 38.3% during the first postoperative year (Tsilimigras et al., 2020). In the study by Di Sandro S et al. (2019), relapse-free survival of 131 BBHCC patients receiving surgery was 34.4, 21.4, 15.3, 6.1, and 2.3% for 2, 4, 6, 8 and 10 years, respectively. In our previous research, outcomes of BBHCC patients receiving TACE improved when treatment was combined with ablation therapy, regardless of whether the patients achieved CR (Zhang et al., 2018). We found that the median time of tumor progression was 10.14 months in the TACE-ablation group, with disease progression rates of 26.0, 52.2, 65.0, and 68.2% at 6, 12, 18, and 24 months, respectively. In the present analysis, the occurrence peak of disease progression occurred approximately around the 9th months; and almost 45% of the patients did not experience tumor progression following the first 3 years after treatment. The superiority in tumor control may be attributed to the fact that the BBHCC patients in our cohort all had CR after treatment. Accordingly, the schedule should be concentrated in the first year posttreatment.

We acknowledge the following limitations of our study. First, our research was retrospectively conducted in a single center for more than 10 years. The next step is to complete multicenter data collection to expand the sample size. Second, the endpoint needs to be defined more specifically, since PFS includes local or regional relapse and metastatic organs beyond the liver. Therefore, the follow-up schedules in these different disease progression types need to be further explored. Third, cost-effectiveness should be analyzed. Last, there are other reported prognostic indicators that were not entered into the RSF tool.

We developed an RSF machine learning method to calculate the disease progression risk per month for BBHCC patients with CR. Afterwards, we established a surveillance strategy that was more effective than the existing surveillance strategies. Our follow-up schedule might shed light on individualized surveillance for BBHCC CR patients.

## Data Availability

The original contribution presented in the study are included in the article/Supplementary Material, further inquiries can be directed to the corresponding author.

## Abbreviations

BCLC, barcelona clinical liver cancer: HCC, hepatocellular carcinoma: BBHCC, BCLC B stage HCC: CR, complete response: TACE, transarterial chemoembolization: RSF, random survival forest: CT, computed tomography: MRI, magnetic resonance imaging: ECIO, European conference on interventional oncology: ESOI, European society of oncologic imaging: PFS, progression-free survival
